# Risk Scoring System for Vancomycin-Associated Acute Kidney Injury

**DOI:** 10.3389/fphar.2022.815188

**Published:** 2022-03-07

**Authors:** Jee Yun Kim, Kyun Young Kim, Jeong Yee, Hye Sun Gwak

**Affiliations:** ^1^ College of Pharmacy and Graduate School of Pharmaceutical Sciences, Ewha Womans University, Seoul, South Korea; ^2^ Department of Pharmacy, Catholic Kwandong University International St. Mary’s Hospital, Incheon, South Korea; ^3^ Division of Nephrology, Department of Internal Medicine, Seoul National University Hospital, Seoul National University College of Medicine, Seoul, South Korea

**Keywords:** nephrotoxicity, acute kidney injury, vancomycin, scoring system, machine learning

## Abstract

Vancomycin-associated acute kidney injury (AKI) remains a major challenge for patients and clinicians. This study aimed to construct a risk scoring system for vancomycin-associated AKI. We retrospectively reviewed medical records of patients who underwent therapeutic drug monitoring for vancomycin from June 2018 to July 2019. We selected possible risk factors for AKI by univariate and multivariable logistic regression analyses and developed a scoring system for vancomycin-associated AKI. Machine learning methods were utilized to predict risk factors for the occurrence of AKI. The incidence of vancomycin-associated AKI was 31.7% among 104 patients included in this study. A bodyweight ≤60 kg (two points), a Charlson comorbidity index ≥3 (two points), a vancomycin trough serum level >15 μg/ml (one point), and concomitant use of ≥6 nephrotoxic agents (two points) were included to construct a risk scoring system based on the coefficient from the logistic regression model. The area under the receiver operating characteristic curve (AUROC) (mean, 95% confidence interval (CI)) across 10 random iterations using five-fold cross-validated multivariate logistic regression, elastic net, random forest, support vector machine (SVM)-linear kernel, and SVM-radial kernel models was 0.735 (0.638–0.833), 0.737 (0.638–0.835), 0.721 (0.610–0.833), 0.739 (0.648–0.829), and 0.733 (0.640–0.826), respectively. For total scores of 0–1, 2–3, 4–5, 6–7, the risk of vancomycin-associated AKI was 5, 25, 45, and 65%, respectively. Our scoring system can be applied to clinical settings in which several nephrotoxic agents are used along with vancomycin therapy.

## Introduction

Vancomycin (VCM) is used for the treatment of severe infections such as sepsis, endocarditis, osteomyelitis, or meningitis caused by methicillin-resistant Staphylococcus aureus (Deresinski, 2009). To achieve optimal efficacy and avoid nephrotoxicity of this drug, therapeutic drug monitoring (TDM) is recommended (Rybak et al., 2020). VCM-associated nephrotoxicity such as acute kidney injury (AKI) is thought to be related to the serum trough level of the drug and/or the area under the curve (AUC) measured in the first 24 h of VCM use (AUC0-24 h) (Bosso et al., 2011; van Hal et al., 2013; Finch et al., 2017; Ghasemiyeh et al., 2020; Poston-Blahnik and Moenster, 2021). Furthermore, concomitant use of nephrotoxic agents along with VCM has been reported to increase the risk of nephrotoxicity of the VCM (Rutter et al., 2017; Gyamlani et al., 2019; Qin et al., 2020). VCM-associated AKI has been reported to worsen patients’ clinical outcomes such as the length of hospital stay, mortality, treatment failure, or economic burden (Wang et al., 2012; Jeffres, 2017). Although many attempts have been made to reduce the incidence of nephrotoxicity associated with the use of VCM, the incidence and severity of nephrotoxicity caused by VCM use are still difficult to predict (Mehta et al., 2015; Filippone et al., 2017).

To predict the AKI risk associated with VCM use, previous studies have identified risk factors for AKI associated with VCM use including sex, age, weight, height, race, renal function, and/or concomitant use of nephrotoxic agents (Finch et al., 2017; Poston-Blahnik and Moenster, 2021; Qin et al., 2020; Gyamlani et al., 2019; Rutter et al., 2017; Mehta et al., 2015; Filippone et al., 2017; See et al., 2021; O'Donnell et al., 2018). However, there are few predictive models for VCM-associated AKI risk that have used machine learning methods other than logistic regression analysis (Imai et al., 2020). Therefore, in this study, we aimed to construct a predictive model for AKI risk due to the VCM use using various machine learning methods and to develop a new scoring system for VCM-associated AKI risk in patients receiving VCM treatment.

## Materials and Methods

### Study Population

We retrospectively reviewed the electronic medical records of patients who were admitted to a university hospital in South Korea from June 2018 to July 2019. The included patients were those who were treated with intravenous VCM for more than 2 days (48 h) or at least four times with adjustment in the VCM dose by TDM. Those who were younger than 18 years of age or who were admitted to the hospital for less than 48 h or for more than 6 months were excluded. This study was approved by the Institutional Review Board of Catholic Kwandong University International St. Mary’s Hospital (approval no. IS19RISI0040) in accordance with the 1964 Helsinki declaration and its later amendments. The requirement for obtaining informed consent was waived by the Institutional Review Board of Catholic Kwandong University International St. Mary’s Hospital, due to the retrospective nature of this study. This study is registered at the Clinical Research Information Service (approval no. KCT0005260).

### Data Collection

At the time of starting TDM, demographic data of patients including sex, age, weight, and height, diagnosis of chronic kidney disease (CKD) defined by glomerular filtration rate (GFR) < 60 ml/min/1.73 m^2^ for 3 months or more (Levey et al., 2005), status of renal replacement therapy, Charlson comorbidity index (CCI), dose and duration of treatment with VCM, and nephrotoxic agents (Supplementary Table S1) received while on VCM therapy were recorded. TDM was performed with both Bayesian dose optimizing software and the trapezoidal method for calculating the lower/upper limit of AUC_0–24h_/minimum inhibitory concentration (MIC). MIC values of VCM were determined by the broth microdilution method. The serum trough levels of VCM were analyzed by chemiluminescent microparticle immunoassay methods using Architect i2000SR (Abbott Laboratories, North Chicago, IL, United States). As the first sampling of serum levels of VCM was performed after administration of at least four times of VCM, all trough levels of VCM were assumed to be at a steady state. For the repeated measurement of laboratory values, only the first serum level was included in the analysis.

### AKI Definitions

The primary outcome was AKI as defined by the Acute Kidney Injury Network (AKIN) criteria (Mehta et al., 2007). A nephrologist examined the changes in serum creatinine (SCr) in these patients and confirmed AKI or any possible stage of nephrotoxicity induced by VCM according to the AKIN criteria: an increase in SCr of ≥0.3 mg/dl (≥26.4 μmol/L), a percentage increase in SCr of ≥50% (1.5-fold from baseline), or a reduction in urine output (documented oliguria of less than 0.5 ml/kg per hour for more than 6 h) within 48 h of VCM use with no other apparent cause. For patients with CKD or those who underwent any form of renal replacement therapy (hemodialysis, peritoneal dialysis, or continuous renal replacement therapy (CRRT)), both the AKIN criteria and urine output-based criteria were used to confirm the occurrence of AKI.

### Statistical Analysis and Machine Learning Methods

Continuous variables were compared with Student’s t-test. If the variables were not normally distributed as determined by one-sample Kolmogorov-Smirnov and Levene tests, the Mann-Whitney test was performed. The Chi-square test or Fisher’s exact test was used to compare categorical variables. The area under the receiver operator characteristic curve (AUROC) was plotted to determine the cut-off values for predicting AKI. Univariate and multivariate logistic regression analyses with the odds ratio (OR) and adjusted OR (AOR), respectively, were used to identify risk factors for AKI. Statistically significant features in the multivariate analysis were used for the machine learning analysis.

Machine learning algorithms were developed to predict risk factors for AKI occurrence (Kim et al., 2021). Five-fold cross-validated multivariate logistic regression, elastic net, random forest (RF), and support vector machine (SVM) classification models were utilized. All the methods were implemented using the R package caret. For cross-validation, the dataset was randomly divided into five equal subsets. After partitioning one data sample into five subsets, one subset was selected for model validation, while the remaining subsets were used to establish machine learning models. Each cross-validation iteration was repeated 10 times to evaluate the power of the machine learning models. To assess the ability of the constructed models to predict AKI incidence, the AUROC and its 95% confidence interval (CI) of each model were calculated.

All statistical tests were two-sided, and *p* values <0.05 were considered statistically significant. The data were analyzed using Statistical Package for Social Sciences Version 20.0 for Windows (SPSS, Chicago, IL, United States). Machine learning algorithms were constructed using R software version 3.6.0 (R Foundation for Statistical Computing, Vienna, Austria).

## Results

Data were obtained for 788 patients, of whom 684 were excluded; 335 did not have any TDM course, 215 had insufficient records, 35 had duplicate courses, 92 had a hospital length of stay <48 h, and 7 had no serum levels of VCM measured. Therefore, 104 patients were included in this study. The baseline demographic characteristics of the study patients are shown in Table 1. The incidence of VCM-associated AKI was 31.7% (33 of 104 patients). The mean age and body weight of the study population were 63.0 ± 15.6 years and 63.0 ± 14.7 kg, respectively.

**TABLE 1:** Association of patient characteristics with AKI.

Characteristics	AKI (*n* = 33)	Non-AKI (*n* = 71)	*p*
Sex			0.587
Male	20 (60.6)	39 (54.9)	
Female	13 (39.4)	32 (45.1)	
Age (years)			0.455
<65	16 (48.5)	40 (56.3)	
≥65	17 (51.5)	31 (43.7)	
Bodyweight (kg)			0.010
≤60	21 (63.6)	26 (36.6)	
>60	12 (36.4)	45 (63.4)	
Body mass index (kg/m^2^)			0.261
<18.5	7 (21.2)	9 (12.7)	
≥18.5	26 (78.8)	62 (87.3)	
Chronic kidney disease			0.914
Yes	3 (9.1)	6 (8.5)	
No	30 (90.9)	65 (91.5)	
Renal replacement therapy^a^			0.579
Yes	5 (15.2)	8 (11.3)	
No	28 (84.8)	63 (88.7)	
Heart failure			0.529
Yes	4 (12.1)	12 (16.9)	
No	29 (87.9)	59 (83.1)	
ICU admission			0.464
Yes	17 (51.5)	42 (59.2)	
No	16 (48.5)	29 (40.8)	
Charlson comorbidity index			0.001
0–2	11 (33.3)	49 (69.0)	
≥3	22 (66.7)	22 (31.0)	
Hospital length of stay (days)			0.210
≤45	25 (75.8)	45 (63.4)	
>45	8 (24.2)	26 (36.6)	
Creatinine (mg/dl)			0.847
<1.2	25 (75.8)	55 (77.5)	
≥1.2	8 (24.2)	16 (22.5)	
eGFR (ml/min/1.73 m^2^)			0.740
<30	4 (12.1)	7 (9.9)	
≥30	29 (87.9)	64 (90.1)	
Vancomycin trough level (mcg/ml)			0.045
≤15	14 (43.8)	46 (64.8)	
>15	18 (54.5)	25 (35.2)	
AUC_0–24h_/MIC (mcg*hr/ml)			0.141
≤800	32 (97.0)	71 (100)	
>800	1 (3.0)	0 (0)	
Total vancomycin dose (g)			0.118
<3	33 (100)	66 (93.0)	
≥3	0 (0)	5 (7.0)	
Vancomycin treatment days			0.301
≤7	5 (15.2)	6 (8.5)	
>7	28 (84.8)	65 (91.5)	
Number of nephrotoxic agents during vancomycin treatment			0.019
0–5	10 (30.3)	39 (54.9)	
≥6	23 (69.7)	32 (45.1)	
Amikacin			0.136
Yes	2 (6.1)	13 (18.3)	
No	31 (93.9)	58 (81.7)	
Colistin			0.093
Yes	3 (9.1)	1 (1.4)	
No	30 (90.9)	70 (98.6)	
Contrast media			
Yes	11 (33.3)	30 (42.3)	0.386
No	22 (66.7)	41 (57.7)	
Furosemide			0.014
Yes	26 (78.8)	38 (53.5)	
No	7 (21.2)	33 (46.5)	
Piperacillin-tazobactam			0.015
Yes	19 (57.6)	23 (32.4)	
No	14 (42.4)	48 (67.6)	

Data are presented as number (%). AUC_0–24h_, area under the curve from 0 to 24 h; MIC, minimum inhibitory concentration; eGFR, estimated glomerular filtration rate.

a Renal replacement therapy includes hemodialysis, peritoneal dialysis, and continuous renal replacement therapy.

In the univariate analysis, significant factors for AKI were a bodyweight of 60 kg or less, a CCI of three or higher, concomitant use of six or more nephrotoxic agents, and a serum trough level of VCM of higher than 15 μg/ml with OR values (95% CI) of 3.03 (1.28–7.14), 4.46 (1.85–10.75), 2.80 (1.17–6.74), and 2.37 (1.01–5.54), respectively (Table 2). Among individual nephrotoxic agents, furosemide and piperacillin-tazobactam significantly increased the AKI.

**TABLE 2:** Univariate and multivariable analyses and score.

Predictors	Unadjusted OR (95% CI)	Adjusted OR (95% CI)	Score
Bodyweight (kg) ≤60	3.03 (1.28–7.14)	3.33 (1.25–8.84)^a^	2
Charlson comorbidity index ≥3	4.46 (1.85–10.75)	3.78 (1.44–9.92)^b^	2
Number of nephrotoxic agents during vancomycin treatment ≥6	2.80 (1.17–6.74)	3.18 (1.14–8.88)^a^	2
Serum trough level of vancomycin (mcg/ml) >15	2.37 (1.01–5.54)	2.00 (0.76–5.26)	1

OR, odds ratio; CI, confidence interval.

a *p* < 0.05.

b *p* < 0.01.

Using the significant features from the univariate analysis in addition to age and sex, we performed multivariate analysis and machine learning analysis. A bodyweight of 60 kg or less, a CCI of three or higher, and concomitant use of six or more nephrotoxic agents remained significant factors after the multivariate analysis with AOR values (95% CI) of 3.33 (1.25–8.84), 3.78 (1.44–9.92), and 3.18 (1.14–8.88), respectively (Table 2). The AUROC of the multivariate analysis was 0.761 (0.665–0.846). The AUROC values (mean, 95% CI) across 10 random iterations using five-fold cross-validated multivariate logistic regression, elastic net, RF, SVM-linear kernel, and SVM-radial kernel models were 0.735 (0.638–0.833), 0.737 (0.638–0.835), 0.721 (0.610–0.833), 0.739 (0.648–0.829), and 0.733 (0.640–0.826), respectively.

**FIGURE 1:**
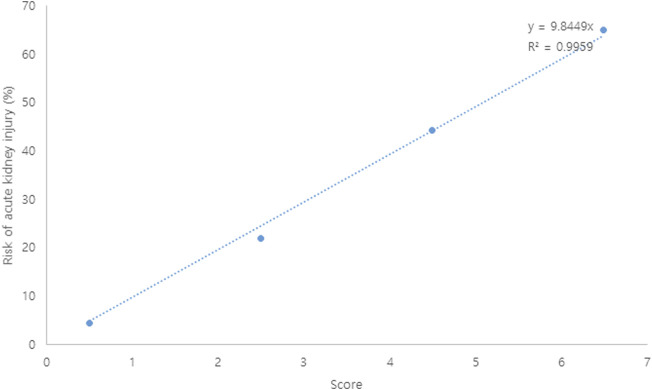
Risk (%) of vancomycin-associated acute kidney injury according to scores.

A risk scoring system was constructed with the selected features from the univariate analysis. Each coefficient from the logistic regression model was divided by the smallest one and rounded to the nearest integer. A bodyweight ≤60 kg (two points), a CCI ≥3 (two points), a VCM trough serum level >15 μg/ml (one point), and concomitant use of ≥6 nephrotoxic agents (two points) were incorporated into the risk scoring system for AKI. Patients with 0–1, 2–3, 4–5, and 6–7 points had about 4.5, 21.9, 44.4, and 64.3% risk of AKI (Table 2). As shown in Figure 1, each point increase resulted in a 10% increase in AKI risk. The AUROC value of the constructed scoring system was 0.769 (95% CI: 0.674–0.863, *p* < 0.001).

## Discussion

In this study, the number of nephrotoxic agents used concomitantly with VCM, disease severity measured by the CCI, and actual patient bodyweight were significant risk factors associated with the occurrence of VCM-associated AKI.

Known risk factors for VCM-associated AKI were patient demographics (age, BMI), VCM dose (>4 g/day), VCM treatment duration (>7 days), serum trough level (>15 μg/ml), comorbidities (e.g., chronic kidney disease and heart failure), and nephrotoxic agents (e.g., aminoglycosides, colistin, contrast media, and diuretics) (Bamgbola, 2016; Karimzadeh et al., 2017; Blair et al., 2021; Higashi et al., 2021; Suzuki et al., 2021). To simplify the risk scoring system, we used CCI and the number of nephrotoxic agents rather than each comorbidity and nephrotoxic agent. For disease severity, we found that as the CCI increased, the risk of AKI also increased. Studies have reported that there were significant differences in the clinical outcomes of patients treated with VCM according to CCI scores (Covert et al., 2020). The CCI incorporates various states such as age, cancer, chronic heart failure, chronic obstructive pulmonary disease, or diabetes mellitus (Charlson et al., 1987). Based on our results, we can stratify the patient population and differentiate the VCM treatment based on CCI for better efficacy and safety.

Regarding bodyweight, a bodyweight ≤60 kg was significantly associated with an increased incidence of AKI. When considering that higher exposure of VCM (e.g., dose, trough concentration, and AUC) was related to VCM-associated AKI (Wong-Beringer et al., 2011; Rostas et al., 2014; Zasowski et al., 2017), the lower the weight, the higher the risk of overdose. This can be also explained by the estimation of glomerular filtration rate (eGFR) as determined using the Modification of Diet in Renal Disease (MDRD) formula (Levey et al., 1999) and the nature of our study population. According to the MDRD eGFR formula, glomerular filtration is negatively related to the serum creatinine level, age, and sex. In this study, the patient group with a bodyweight ≤60 kg had higher proportions of females, those ≥65 years, and those with an intensive care unit admission than the patient group with a bodyweight >60 kg. Therefore, the patient group with a bodyweight ≤60 kg had a slower GFR than the patient group with a bodyweight >60 kg, which can increase the risk of AKI. This result is in line with the findings of previous studies, which reported that a lower body mass index (BMI) is a risk factor for AKI in Asian patients (Liu et al., 2018).

The serum trough levels of VCM were significantly associated with AKI in the univariate analysis; however, statistical significance was not found in the multivariate analysis. We included the serum trough level as a risk factor for the scoring system since higher serum trough levels of VCM were associated with an increased incidence of nephrotoxicity (Bosso et al., 2011; van Hal et al., 2013; Rybak et al., 2020).

The AUROC values indicated the favorable performance of these models, regardless of the machine learning method used in this study (higher than 0.7). The elastic net is a penalized linear regression model that combines the penalties of the lasso and ridge methods (Zou and Hastie, 2005). RF is an ensemble method that increases the diversity by using a random subset of available features at each node and provides a more accurate prediction than a single decision tree (Breiman et al., 1984; Breiman, 2001; Hastie et al., 2009). In this study, SVMs were implemented using linear and radial basis function kernels. Linear kernel SVMs have a single tuning parameter, C, which is the cost parameter of the error term, whereas radial kernel SVMs have an additional hyperparameter, sigma, which determines the width for Gaussian distribution (Cortes and Vapnik, 1995; Hastie et al., 2009).

This study has several limitations. First, it has a small sample size with single-center cohort data and retrospective design. Second, we did not evaluate the severity and prognosis of AKI. Third, we did not perform the renal biopsy for identifying if it is a dose-independent acute tubulointerstitial nephritis or a dose-dependent renal tubular injury. Fourth, AKI was defined according to AKIN (Mehta et al., 2007) rather than other criteria (e.g., RIFLE (Bellomo et al., 2004), KDIGO (Kidney Disease: Improving, 2012), and ASHP (Rybak et al., 2009)), which may cause heterogeneity and need cautions in the interpretation. However, our results have strengths in the methodologies used to develop a scoring system and incorporate various types of possible nephrotoxic agents. Moreover, we included patients with CKD and found that their renal impairment status was not significantly related to the risk of AKI. The broad spectrum of diseases in our studied population, which included patients with CKD, is notable in that previous studies have mainly dealt with patients with CKD patients alone or excluded these renally impaired populations (Liu et al., 2018; Freitas et al., 2020; Gaggl et al., 2020; Imai et al., 2020). Thus, our scoring system for AKI can be generalized to patients with various types and severities of diseases.

## Conclusion

In this study, we aimed to develop an optimal scoring system for predicting VCM-associated AKI using machine learning methods. To the best of our knowledge, this is the first study to propose a risk scoring system for VCM-associated AKI in Korean patients undergoing TDM with concomitant use of various nephrotoxic agents. Moreover, our studied patients had varied disease types such as sepsis, osteomyelitis, central nervous system infections, pneumonia, and CKD of various stages and were undergoing renal replacement therapies. Thus, our results can be used to develop guidelines or effective treatment strategies for VCM use in adult patients who are receiving nephrotoxic agents. Further studies using a larger population are needed to confirm our results.

## Data Availability

The raw data supporting the conclusions of this article will be made available by the authors, without undue reservation.
