# Supplementary material for: Risk Scoring System for Vancomycin-Associated Acute Kidney Injury
Source: Front Pharmacol. 2022 Mar 7;13:815188. doi: 10.3389/fphar.2022.815188 (PMC8940364; doi:10.3389/fphar.2022.815188)
Supplement: Supplementary file 1 [file Table1.DOCX]

Supplementary Material

# Supplementary Table 1. List of nephrotoxic agents

|  |  |  |  |  |  |  |
| --- | --- | --- | --- | --- | --- | --- |
|  | List of nephrotoxic agents | | | | |  |
|  |  |  |  |  |  |  |
|  | Aceclofenac | Cefpodoxime | Etodolac | Mannitol | Rifampin |  |
|  | Acetaminophen | Cefprozil | Everolimus | Meclofenamate | Ritonavir |  |
|  | Acyclovir | Ceftaroline | Fenoprofen | Mefenamic acid | Salsalate |  |
|  | Acyclovir | Ceftazidime | Fluoxetine | Meloxicam | Sirolimus |  |
|  | Albumin | Ceftibuten | Flurbiprofen | Mesalamine | Streptomycin |  |
|  | Allopurinol | Ceftriaxone | Foscarnet | Methotrexate | Streptozocin |  |
|  | Amikacin | Cefuroxime | Fosinopril | Methylephedrine | Sulfadiazine |  |
|  | Amoxicillin | Celecoxib | Furosemide | Milrinone | Sulfasalazine |  |
|  | Amphotericin B | Cephalexine | Furosemide | Mitomycin C | Sulindac |  |
|  | Arbekacin | Cidofovir | Ganciclovir | Moexipril | Tacrolimus |  |
|  | Aspirin | Cisplatin | Gemcitabine | Nabumetone | Teicoplanin |  |
|  | Azacitidine | Clindamycin | Gentamicin | Nafcillin | Telmisartan |  |
|  | Azilsartan | Colistin | Hetastarch | Naproxen | Tenofovir |  |
|  | Benazepril | Contrast media | Hydroxyethylstarch | Neomycin | Tobramycin |  |
|  | Bevacizumab | Cyclosporine | Ibuprofen | Norepinephrine | Tolmetin |  |
|  | Bumetanide | Dapsone | Ifosfamide | Olmesartan | Torsemide |  |
|  | Candesartan | Dexlansoprazol | Imipenem | Oxaprozin | Tramadol |  |
|  | Captopril | Diclofenac | Immunoglobulin | Pantoprazole | Tramadol |  |
|  | Carboplatin | Diflunisal | Indinavir | Paracetamol | Trandolapril |  |
|  | Cefaclor | dobutamine | Indomethacin | Perindopril | Trimethoprim-sulfamethoxazole |  |
|  | Cefadroxil | Dopamine | Irbersartan | Phenylephrine |  |  |
|  | Cefazolin | Dopamine | Isoproterenol | Piperacillin/tazobactam | Valacyclovir |  |
|  | Cefbuperazone | Enalapril | Kanamycin | Piroxicam | Valganciclovir |  |
|  | Cefdinir | Entecavir | Ketoprofen | Polymyxin B | Valsartan |  |
|  | Cefditoren | Epinephrine | Ketorolac | Propacetamol | Valsartan |  |
|  | Cefepime | Eprosartan | Lansoprazol | Quinapril | Vancomycin |  |
|  | Cefixime | Esomeprazole | Lisinopril | Ramipril | Vasopressin |  |
|  | Cefotaxime | Ethacrynic acid | Lithium | Rifampicin | Zaltoprofen |  |
|  |  |  | Losartan |  | Zonisamide |  |
|  |  |  |  |  |  |  |
|  |  |  |  |  |  |  |
|  |  |  |  |  |  |  |
